# Age and gender variations in the cone-beam computed tomographic location of mandibular canal: Implications for mandibular sagittal split osteotomy

**DOI:** 10.4317/medoral.22969

**Published:** 2019-06-25

**Authors:** Mohammad H. Al-Shayyab, Khalid Qabba’ah, Firas Alsoleihat, Zaid H. Baqain

**Affiliations:** 1 MSc (Eng.), FFD RCS (Ire.). Assistant Dean, Associate Professor in Oral and Maxillofacial Surgery, Department of Oral and Maxillofacial Surgery, Oral Medicine and Periodontology, School of Dentistry, The University of Jordan, Amman, Jordan 11942; 2 BDS. Resident in Oral and Maxillofacial Surgery, Department of Oral and Maxillofacial Surgery, Oral Medicine and Periodontology, School of Dentistry, The University of Jordan, Amman, Jordan 11942; 3 BDS, PhD. Assistant Dean. Associate Professor in the Department of Conservative Dentistry, Department of Conservative Dentistry, School of Dentistry, The University of Jordan, Amman, Jordan 11942; 4 MSc (Eng.), FDS RCS (Eng.). Vice president, Professor in Oral and Maxillofacial Surgery, Department of Oral and Maxillofacial Surgery, Oral Medicine and Periodontology, School of Dentistry, The University of Jordan, Amman, Jordan 11942

## Abstract

**Background:**

Mandibular sagittal split osteotomy (MSSO) may incur unfavorable split and sensorineural injuries. Knowledge of the anatomic location of the mandibular canal (MC) and bone thickness in the region of interest for MSSO, and the possible variations by age and gender can assist in avoiding such complications. Purpose: To study the location of the MC and bone thickness in the region of MSSO by cone-beam computed tomography (CBCT) radiographs and to evaluate the possible variations by age and gender in a Jordanian population.

**Material and Methods:**

This retrospective radio-anatomical study examined all CBCT radiographs for patients treated over three years at the University of Jordan Hospital, Amman, Jordan. Distances from the MC to the cortical external surfaces and MC diameter (MCD) were measured by a reliable observer at three predetermined regions for MSSO: region (A) [mandibular foramen area], region (B) [mandibular angle area] and region (C) [directly mesial to the second molar]. Gender and age differences in all measurements were then compared using non-parametric Mann-Whitney U test.

**Results:**

The final study radiographs comprised a total of 202 CBCT belonged to a cohort of 202 subjects; 91 males (45.1%) and 111 (54.9%) females, with mean age (± SD) of 42.94 ± 18.54 years (range 18–90 years). Whereas only the bone thickness superior, buccal and inferior to MC in regions (B) and (C), and MCD in the three regions exhibited significant (*p*< 0.05) gender differences, all measured distances exhibited statistically significant (*p*< 0.05) differences between young and adult patients.

**Conclusions:**

The location of MC and bone thickness in the region of MSSO were significantly variable according to age, but exhibited sexual diamorphism only in regions (B) and (C). This fundamental knowledge should be considered during MSSO planning.

** Key words:**Cone-Beam Computed Tomography, mandibular canal, mandibular sagittal split osteotomy.

## Introduction

Mandibular sagittal split osteotomy (MSSO) is the standard orthognathic surgical procedure commonly indicated for correction of mandibular deformities, such as prognathism, retrognathism and asymmetry (1). This technique was first popularized by Trauner and Obwegeser in 1957 (2). Since then, it had undergone several modifications aiming at minimizing the risk of important complications, such as unfavorable fractures (bad splits) and sensorineural disturbances in the lower lip, gingiva and chin region (3,4). Such complications can negatively affect the recovery and daily life of patients submitted to orthognathic surgery (5), and seem to be clearly related to the positioning and depth of the osteotomy cuts during MSSO (4). Therefore, the osteotomy design should be decided based on the bone thickness and mandibular morphology in the vicinity of the inferior alveolar nerve (IAN) (6,7).

Ample literature (8-11) investigated mandibular morphology using various anatomical and radiological methods, and reported variations possibly related to ethnicity, age, gender, dental status and dentofacial skeletal relationships. However, very few published English literature (12,13) investigated the bone thickness and mandibular morphology in the vicinity of the IAN for MSSO using advanced imaging, and reported no significant variations among patients with different dentofacial skeletal relationships. To the authors’ knowledge, no published English literature investigated such variations by both age and gender for MSSO. Hence, the aim of this study was to investigate, through cone-beam computed tomography (CBCT) radiographs, the anatomic location of the mandibular canal (MC) and bone thickness in the region of MSSO, and to demonstrate any possible variations by age and gender in a Jordanian population; it has been investigated for the first time in this population.

## Material and Methods

-Study subjects

This retrospective radio-anatomical study reviewed all available CBCT radiographs at the University of Jordan Hospital (UJH), Amman, Jordan. The UJH is a referral centre located at the capital city of Amman and provides a comprehensive health service for more than half a million people annually. The Research Ethics Committee at UJH approved this study (reference number 10/2018/21807), and the study was conducted in full accordance with the Declaration of Helsinki. A total of 233 CBCT radiographs primarily satisfied the inclusion criteria and belonged to 233 Jordanian subjects referred to and treated in the Department of Dentistry at the UJH for various dentofacial problems, between September 2015 and August 2018. Only CBCT radiographs showing an optimal viewing and diagnostic quality for patients aged ≥ 18 years and presented with a complete set of mandibular posterior teeth and facial symmetry were included in this study. Those showing evidence of pathological lesions, missing or impacted teeth, or previous mandibular surgery as well as any developmental anomalies altering the position of the teeth, the MC or other concerned landmarks were excluded.

-CBCT radiographs

A senior radiology technician acquired all CBCT radiographs used in this study, according to the manufacturer’s instructions and a strict, standardized scanning protocol at the UJH. These radiographs were obtained using a CBCT scanner (CS 9300. Carestream Health, Inc., 10622 AL 93 SS 0314, France, 2014), which was made at 60–90 kVp and 2–15 mA at different resolutions, with an exposure time of 4–16 s and a voxel size of 90–300 μm depending on the field of view. Analysis of CBCT radiographs were also made at 2 mm slice thickness.

-Calibration and inter-observer reliability

To avoid inter-observer differences, all CBCT radiographs used in this study were evaluated by the same observer [a senior oral and maxillofacial surgery (OMFS) resident]. This observer was calibrated to identify the MC and other concerned landmarks in the region of MSSO using a set of CBCT radiographs not included in the study for the purpose of training. Calibration sessions were presented to the observer by the senior maxillofacial radiologist at the UJH and entailed practical demonstrations and discussions on the identifications of MC and other concerned landmarks and on the method of using the CBCT software to measure distances between these landmarks. This stage was considered successfully completed when the observer demonstrated a proficient capability of identifying the MC and other concerned landmarks and of measuring distances between these landmarks. The observer was then given a set of CBCT radiographs not included in the study to measure all required distances, which were re-measured by the maxillofacial radiologist. The inter-observer reliability was then assessed using the intraclass correlation coefficient (ICC), which was overall ranged from 83.9 to 99.8%, with 95% confidence interval (CI) of 0.592–0.999 for all various measurements. This indicated an excellent level of agreement (*P* < 0.001) between the observer and the maxillofacial radiologist, and would suggest that the observer was reliable in examining the experimental CBCT radiographs.

-CBCT analysis and evaluation techniques

**Figure 1:**
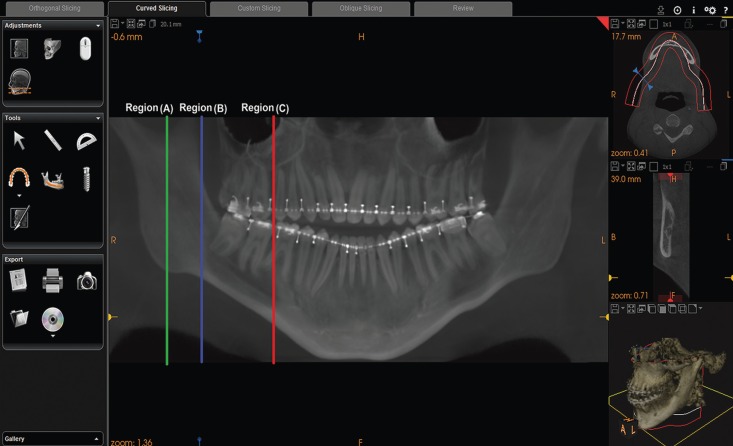
Reconstructed panoramic view showing the lines at which all measurements were obtained at the level of three specific regions of interest for mandibular sagittal split osteotomy. Region (A): The mandibular foramen (MF) area; Region (B): The transitional area between the mandibular ramus and body; Region (C): The area directly mesial to the second mandibular molar.

When conducting CBCT analysis, the calibrated observer was blinded to any other patients’ details. All CBCT radiographs were examined in standard viewing conditions by enhancing the software’s image processing tool for adjusting the brightness and contrast values to ensure optimal visualization. In addition, the software allowed the axial, cross-sectional, sagittal, and panoramic reconstructions, which were pre-oriented and used as necessary to identify the location of the MC and other concerned landmarks. For each patient, the cross-sectional scan was taken and valid measurements were ensured by obtaining these measurements through the same reproducible lines at the level of three specific regions of interest for MSSO (Fig. 1), as recently described (7):

• Region (A): the mandibular foramen (MF) area (the first view in which the foramen was detected).

• Region (B): the transitional area between the mandibular ramus and body (mandibular angle area) [obtained through a straight line that was crossing the MC and tapping the most anterior border of the ramus].

• Region (C): the area directly mesial to the second mandibular molar.

**Figure 2:**
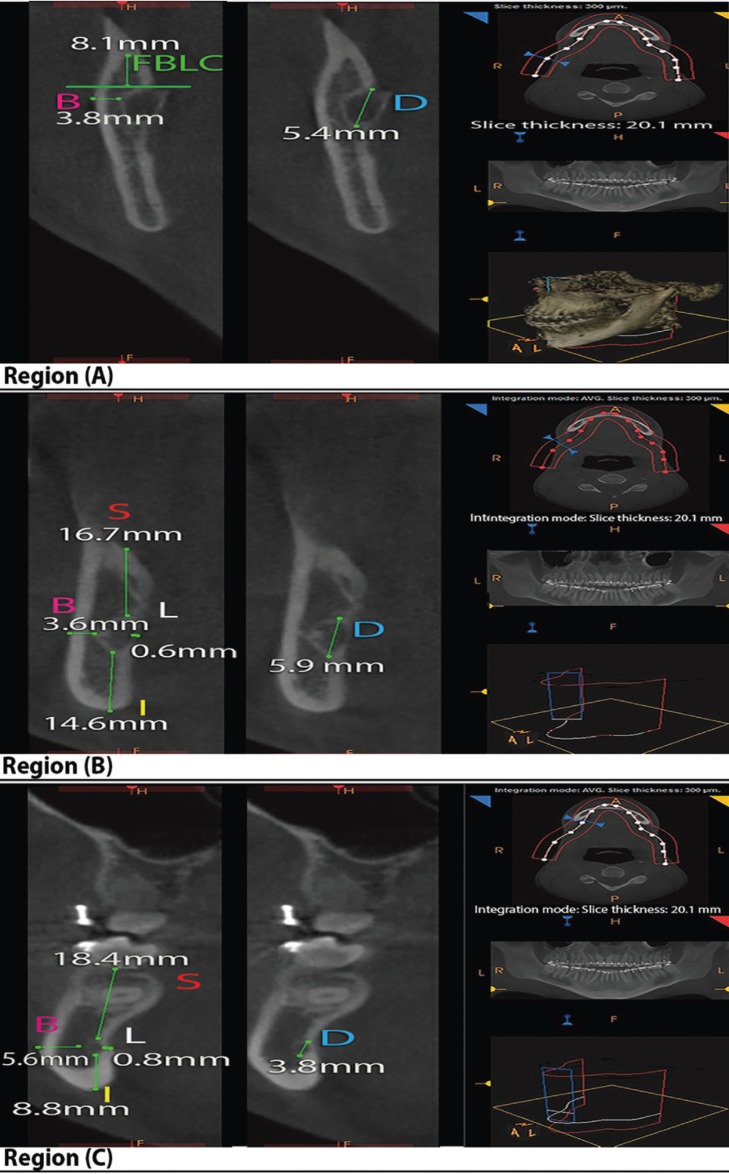
Reconstructed cross-sectional views with the software ruler measuring distances from the external surface of mandibular canal (MC) to the cortical external surfaces. Region (A): The mandibular foramen (MF) area; Region (B): The transitional area between the mandibular ramus and body; Region (C): The area directly mesial to the second mandibular molar; FBLC: Fusion between the buccal and lingual cortices above MF; S: The external surface of the alveolar crest; B: The external surface of the buccal cortical plate; I: The external surface of the inferior border of the mandible; L: The external surface of the lingual cortical plate; D: Mandibular canal diameter.

In the reconstructed cross-sectional views (Fig. 2), the software ruler was enhanced to measure distances (in millimeters) between the MC and specific mandibular landmarks used in recent studies (7,11,12): in region (A), the distance between the superior cortex of MF and the fusion between the buccal and lingual cortices (FBLC) above MF [MC-FBLC], and between the outer surface of MC and the external surface of the buccal (B) cortical plate [MC-B]; in regions (B) and (C), distances between the outer surface of MC and the external surface of the superior (S) [MC-S], lingual (L) [MC-L], inferior (I) [MC-I] and B [MC-B] cortical plates of mandibular bone; and in the three regions, the largest diameter of the MC [MCD] (Fig. 2).

All measurements for each subject were assessed on the right and left sides, and hence, 466 CBCT radiographs of the MC and of the bone thickness between the MC and other concerned landmarks were primarily analyzed. Patients’ file number, age, and gender were then recorded, and according to their age, they were divided into two groups; young (18–40 years), and adult (> 40 years). This classification border considered the fact, demonstrated in a recent study (8) on the same population, that mandibular growth and remodeling in the vicinity of the IAN goes on until the bone has reached the adult size (40 years), following which the deposition and resorption process is imbalanced and results in a significantly decreased ramus measurements. It would also create comparable cohorts in this study.

-Intra-observer reliability and power analysis

The same calibrated observer was asked to measure all distances in the included CBCT images twice in a two-month interval. The ICC was then used to assess the level of intra-observer agreement between the two measurement sessions, and hence intra-observer reliability. The statistical software package G*Power version 3.1.5 (Franz Faul, Universität Kiel, Kiel, Germany, 1992) was used to calculate the statistical power of this study. This was performed retrospectively, and hence post-hoc power analysis was conducted and a Mann-Whitney U test of two independent groups was set as a statistical test to perform power analyses for age and gender comparisons at an effect size of 0.5 (Cohen’s medium effect size), α error probability of 0.05, and a sample size of 202 subjects valid for age and gender analyses; 93 young subjects versus 109 adults, and 91 males versus 111 females.

-Statistical analysis

The Statistical Package for Social Sciences for Windows version 19 (SPSS, Chicago, IL, USA) was used to analyze the collected data, and the statistical significance was based on probability values of < 0.05. Descriptive statistics were produced for the included subjects and their measurements, overall and within the groups. To obtain valid results, data was ensured to pass the assumptions required for parametric and non-parametric tests. The Shapiro-Wilk test was initially used to assess the normality of the collected data and indicated non-parametric tests to compare mean ranks, overall and within the groups. As such, differences in the measurements between the right and left hemi-mandibles were analyzed using Wilcoxon signed-ranks test. Gender and age differences in all measurements were also compared using Mann-Whitney U test, overall and within the same group.

## Results

-Study subjects

Out of the 233 CBCT radiographs primarily satisfied the inclusion criteria for this study, 31 were further excluded; seven belonged to subjects aged < 18 years, eight showed poor diagnostic quality and mandibular asymmetry and 16 showed evidence of pathological lesions, missing or impacted teeth, or previous mandibular surgery as well as developmental disturbances altering mandibular morphology. The final study radiographs comprised a total of 202 CBCT belonged to a cohort of 202 subjects; 91 males (45.1%) and 111 (54.9%) females, with mean age (± SD) of 42.94 ± 18.54 years (range 18–90 years). Descriptive statistics of the final study cohort are presented in  Table 1, overall and within the same gender and age group.

**Table 1:**
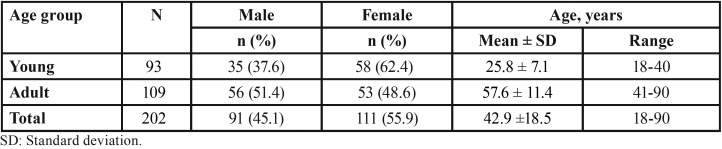
Descriptive statistics of the study groups (age and sex) (N=202).

-Intra-examiner reliability and power analysis

Repeated CBCT measurements by the same reliable observer indicated a significant (*P* < 0.001) level of intra-observer agreement between the two measurement sessions; an overall ICC ranging from 95.5% to 99.4%, with 95% confidence interval (CI) of 0.941–0.995 for all various measurements ( Table 2). Therefore, the intra-observer difference was random and statistically not significant. Analysis computation for this study also yielded 97.0% statistical power for age and gender analyses; thus, demonstrating that such retrospective study was very adequate for detecting age and gender statistical variations.

**Table 2:**
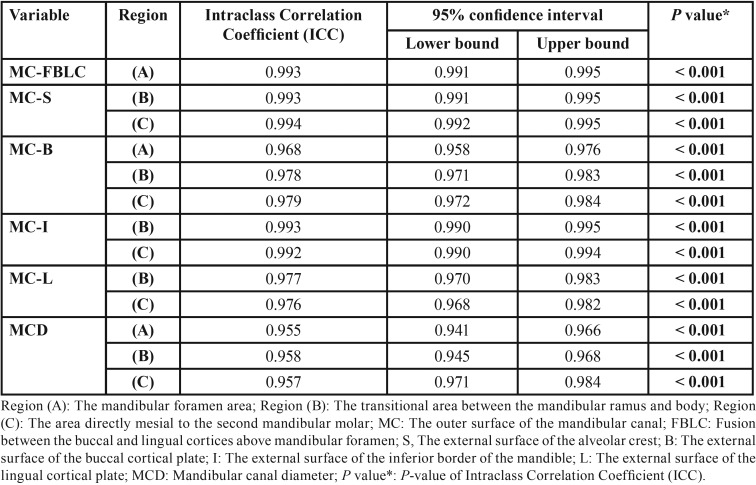
Intra-observer reliability.

-Comparison of measurements between the right and left hemi-mandibles

In the three regions of interest for BSSO, there were no statistical significant (*P* > 0.05) differences between measurements in the right and left hemi-mandibles. Therefore, measurements from both sides have been averaged and then used in later analyses.

-Comparison of the distances between males and females

The average, minimum and maximum measurements and standard deviation among males and females are presented in  Table 3, overall and within the same age group. Whereas the MC-FBLC, MC-B in region (A) and MC-L distances were not significantly (*P* > 0.05) different between males and females, the MC-S in region (C) and MCD in the three regions were significantly longer in males than in females, overall [MC-S (region C): U= 3505, *P* < 0.001; MCD: U = 3514.5-3532.5, *P* < 0.001] and within the same age group [young MC-S (region C): U= 476.0, *P* < 0.001; adult MC-S (region C): U=1109.0, *P* < 0.05; young MCD: U = 597.5-619.5, *P*=0.001-0.002; adult MCD: U= 919.5-923.5, *P*= 0.001]. However, The average MC-S and MC-I in region (B), and MC-B in regions (B) and (C) distances exhibited significant higher measurements in males than in females within a specific age group [young MC-S (region B): U= 561, *P* < 0.001; adult MC-I (region B): U= 855.0, *P* < 0.001; adult MC-B (region B): U= 952.0, *P* < 0.05; adult MC-B (region C): U= 744.5, *P* < 0.001]. By contrast, the average MC-I distance in region (C) exhibited significant higher measurements in females than in males only among the young age group (U= 310.0, *P* < 0.001) ( Table 3).

**Table 3:**
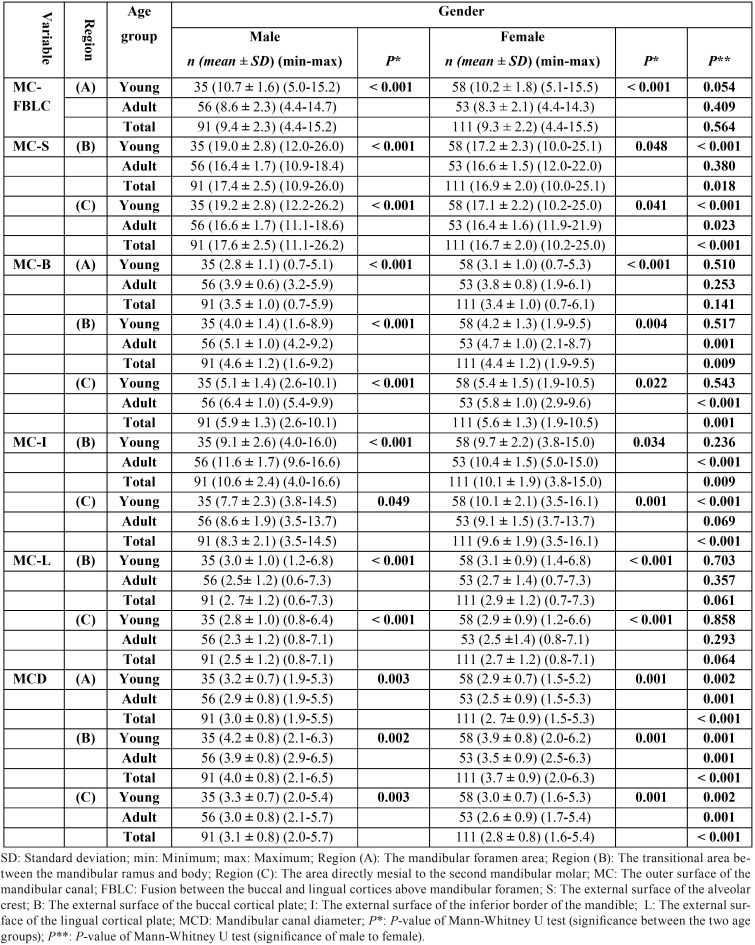
Comparison of the average distances at the level of three specific regions of interest for mandibular sagittal split osteotomy (BSSO); between the two age groups within the same gender, and between males and females, overall and within the same age group (N=202).

-Comparison of the distances between young and adult patients

The average, minimum and maximum measurements and standard deviation among young and adult age groups are presented within the same gender in  Table 3, and overall in  Table 4; adults exhibited a decrease in the average values of MC-FBLC, MC-S, MC-L, and MCD, and an increase in the average values of MC-B and MC-I, compared with young patients. Mann-Whitney U test generally indicated that such age differences in these average distances were statistically significant, within the same gender (male: U= 294.5-738.5, *P*=0.000-0.049; female: U= 621.5-1205.5, *P* =0.000-0.048) ( Table 3), and overall (U= 2067.0-4294.0, *P*= 0.000-0.049) ( Table 4).

**Table 4:**
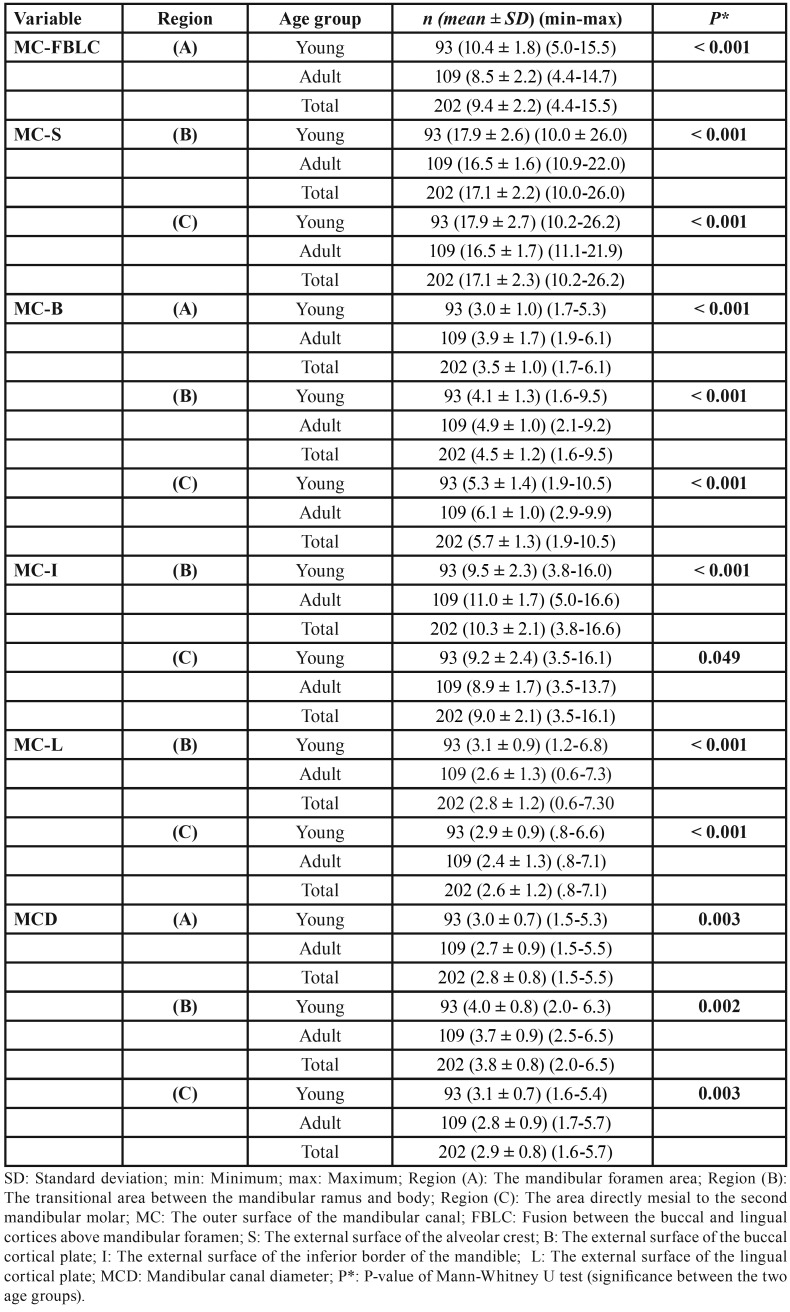
Comparison of the average distances at the level of three specific regions of interest for mandibular sagittal split osteotomy (BSSO); between the two age groups in the entire cohort (N=202).

## Discussion

Knowledge of the anatomic location of the MC and bone thickness in the region of interest for MSSO and the possible variations by age and gender can help oral and maxillofacial surgeons to decide on the safest osteotomy design that is tailored to specific age and sex (7,11,14), and may assist in evaluating the risk of bad split and the degree and extent of IAN injury resulting from both direct and indirect surgical trauma (15). CBCT is well suited for anatomic structures in the maxillofacial region (16,17), and has several advantages over conventional CT and plain films: reduced effective radiation dose, fewer artifacts, and high accuracy and reproducibility (18).

In this study, the MC-FBLC, MC-B and MCD distances measured in region (A) are of clinical relevance to the horizontal osteotomy cut in MSSO, whereas the MC-B, MC-S, MC-L, MC-I and MCD distances measured in regions (B) and (C) are of clinical relevance to the sagittal and vertical osteotomies of MSSO. In this study, the MC generally presented a variable diameter that increased in region (B) and decreased in region (C) to become close to diameter in region (A), with a significantly longer diameter among adults and males when compared to young patients and females, respectively. The FBLC was significantly located at a mean height of 10.4 mm with a minimum distance of 5 mm in young patients compared with a mean height of 8.5 mm with a minimum distance of 4.4 mm in adults from the MF, with no significant sexual dimorphism. In addition, the MC-B distance in region (A) significantly measured an average distance of 3 mm in young patients compared with an average distance of 3.9 mm in adults, regardless of the gender. Interestingly, the sum values of the vertical and horizontal distances in regions (B) and (C) showed that female mandibles had smaller vertical dimension in region (B) and thinner buccolingual dimension in region (C) than males, but adult mandibles had a smaller vertical dimension with a buccolingual dimension similar to young mandibles. These results are a bit higher but comparable to the CBCT findings of Sekerci and Sahman (11) in a Turkish population and Scomparion *et al.* (7) in a Brazilin population (as expected due to ancestral similarity), and to the findings of Yu and Wong (10) in a Taiwanese population (although unexpected due to ancestral dissimilarity) using 3DCT images; nevertheless the authors did not present any data regarding the comparison of the measurements in terms of age, but presented gender differences that were generally not in corroboration with this study. Noleto *et al.* (13) and Huang and Liao (12) demonstrated no significant variations in Brazilian and Chinese patients, respectively, with different dentofacial skeletal relationships, but reported nearly close measurements (as expected due to ancestral similarity to Brazilians, but unexpected due to ancestral dissimilarity to Chinese); however, the authors did not present any data regarding the comparison of the measurements in terms of age and sex and used the lingula, not the superior cortex of the MF, as a landmark for the measurements what can difficult the comparison with this study.

The findings of this study indicated an absolute positional bilateral symmetry of the MC and bone thickness in the region of MSSO, and this is in agreement with previous studies (7,8,11). As for gender, gender did not always lead to a significant variation in distances measured in this study, and this is in agreement with recent studies (7,11) and with the fact that genetic and environmental factors may also influence bone size and thickness (8,19). However, measurements recorded in this study were significantly variable according to age and in concordance with the fact that the position of the MC in relation to other landmarks is not constant and affected by imbalanced growth and remodeling occurred with advancing age, characterized by thickening of the buccal and inferior aspects of mandibular bone and resorption of the superior and lingual aspects moving the MC in a superior and lingual direction and leaving a decreased amount of cancellous bone, and shorter ramus and mandibular posterior region (8,20,21). Aarabi *et al.* (22) reported that patients with smaller amount of bone in region (A) and shorter rami were more likely to present bad splits in the lingual side of the distal segment during MSSO. A shorter ramus probably leads to a more difficult surgical access and shorter MC-FBLC distance. In addition, the smaller amount of bone in region (A) may contribute to mandible brittleness towards the forces applied during horizontal osteotomy (22). Aarabi *et al.* (22) also reported that patients with a thinner buccolingual distance in regions (B) and (C) presented an increased risk for unfavorable fractures in the distal or proximal segments during MSSO. Thus, the suggestion (6,10,15) that the area between the first and second molars along the crest of the external oblique line is the best location to perform the vertical anterior osteotomy; presumably because of a greater buccal bone thickness, and hence a lower chance of IAN injury and badsplits. In addition, the shorter mandibular posterior region presents more difficult surgical access and may contribute to mandible brittleness towards the forces applied during sagittal and vertical osteotomies (24). Witherow *et al.*, (23) in a study using panoramic radiographs, stated that patients with a mandibular retromolar height of < 2 cm and with a distance between the apices of the last molar tooth and the inferior border of the mandible of < 0.6 cm were at higher risk of developing unfavorable fractures of the lingual plate during MSSO. Furthermore, CBCT studies have shown that the distance between the mandibular canal and the split surface is correlated with the trigeminal somatosensory-evoked potential latency recovery (24). Thus, in light of the aforementioned literature (6,8,10,15,20-23), the findings of the current study indicated a significantly increased chance of IAN injury and unfavorable fractures during MSSO among adults compared with young patients, with a slightly increased chance among females during sagittal and vertical osteotomies of MSSO. Therefore, the current study would suggest that the horizontal mandibular ramus osteotomy has to be performed at an average point not exceeding 9 mm in young patients and 7 mm in adults from the MF, and carried to the depth of the medial surface of the buccal cortex, which would be deeper in young patient than in adults; this would help the surgeon in avoiding a region that could considerably increase the incidence of unfavorable fractures (23). In addition, the sagittal mandibular osteotomy has to be performed more buccal and not deeper in adults and females than in young and female patients; this would help the surgeon in avoiding the IAN and providing adequate lingual bone thickness that could considerably decrease the incidence of unfavorable fractures in the distal or proximal segments (23). Furthermore, it is important to consider using miniplates and monocortical fixation in patients with thinner superior and lingual bone thickness, to avoid the transmission of forces to the lingual plate reducing the risk of unfavorable fractures (19). Our findings also suggest that the vertical mandibular osteotomy has to be carried to the depth of the medial surface of the buccal and inferior mandibular cortices, which would be deeper for the buccal osteotomy in adult and male patients, and for the inferior border osteotomy in adult and female patients; this would make the surgeon reach a depth that could considerably decrease the incidence of bad split and IAN injury during MSSO (15).

Despite the abovementioned findings, this research is not without limitations; it did not account for the possible variations by dentofacial skeletal relationships: although reported (12,13) to be not significant, but should not be ignored in future research to achieve a reliable conclusion. In addition, differences in population and/or measurement techniques could be the reason for the relative discrepancy between this study and other similar studies (7,11-13). Although the current study was based on a sample size that was greater than in previous studies (7,11,12) and yielded 97% statistical power, larger sample sizes from other populations and with clear indications for MSSO are still needed to validate the results of this study and to derive a highly valid ‘evidence’ determining the safest osteotomy design.

In conclusion, the location of MC and bone thickness in the region of MSSO were significantly variable according to age, but exhibited sexual diamorphism only in regions (B) and (C). This fundamental knowledge may help the maxillofacial surgeon to outline an osteotomy design tailored to the patient’s age and sex. Nevertheless, the relative variability of the measurements between this study and other similar studies would stress the important rule of the preoperative analysis of the patient’s own CBCT.
